# Ethanol inhibits dopamine uptake via organic cation transporter 3: Implications for ethanol and cocaine co-abuse

**DOI:** 10.1038/s41380-023-02064-5

**Published:** 2023-06-13

**Authors:** N. J. Clauss, F. P. Mayer, W. A. Owens, M. Vitela, K. M. Clarke, M. A. Bowman, R. E. Horton, D. Gründemann, D. Schmid, M. Holy, G. G. Gould, W. Koek, H. H. Sitte, L. C. Daws

**Affiliations:** 1 Department of Cellular and Integrative Physiology, University of Texas Health Science Center at San Antonio, San Antonio, TX 78229 USA; 2 Center for Physiology and Pharmacology, Medical University of Vienna, 1090 Vienna, Austria; 3 Department of Neuroscience, Faculty of Health and Medical Sciences, University of Copenhagen, Copenhagen, DK-2200, Denmark; 4 Department of Pharmacology, Faculty of Medicine and University Hospital Cologne, University of Cologne, 50931 Cologne, Germany; 5 Department of Cell Systems and Anatomy, University of Texas Health Science Center at San Antonio, San Antonio, TX 78229 USA; 6 Center for Addiction Research and Science, Medical University Vienna, Waehringerstrasse 13 A, 1090 Vienna, Austria; 7 Department of Pharmacology, University of Texas Health Science Center at San Antonio, San Antonio, TX 78229 USA

**Keywords:** Neuroscience, Physiology

## Abstract

Concurrent cocaine and alcohol use is among the most frequent drug combination, and among the most dangerous in terms of deleterious outcomes. Cocaine increases extracellular monoamines by blocking dopamine (DA), norepinephrine (NE) and serotonin (5-HT) transporters (DAT, NET and SERT, respectively). Likewise, ethanol also increases extracellular monoamines, however evidence suggests that ethanol does so independently of DAT, NET and SERT. Organic cation transporter 3 (OCT3) is an emergent key player in the regulation of monoamine signaling. Using a battery of in vitro, in vivo electrochemical, and behavioral approaches, as well as wild-type and constitutive OCT3 knockout mice, we show that ethanol’s actions to inhibit monoamine uptake are dependent on OCT3. These findings provide a novel mechanistic basis whereby ethanol enhances the neurochemical and behavioral effects of cocaine and encourage further research into OCT3 as a target for therapeutic intervention in the treatment of ethanol and ethanol/cocaine use disorders.

## Introduction

Alcohol use disorder (AUD) is the most common substance use disorder (SUD) in the US [1]. Problematic alcohol use contributes significantly to disease burden worldwide and is associated with various adverse economic and social outcomes [2]. Even more concerning, alcohol is often used with other substances [3–10], frequently with cocaine [11–16]. This drug pairing, which often causes emergency hospitalization, is likely more toxic than each substance alone [15, 16]. There is currently no treatment for co-abuse of, and overdose with, alcohol and cocaine, further highlighting the hazards of their concurrent use and underscoring the need to understand its mechanistic basis. Furthering this understanding is necessary to discover new targets for therapeutic intervention in treating AUD, including co-abuse of alcohol and cocaine.

The popularity of co-abuse of alcohol and cocaine could result from an increased “high” compared with taking either drug alone, as well as from a prolongation of cocaine-induced euphoria [16–18], possibly because increases in dopamine (DA), norepinephrine (NE), and serotonin (5-HT) may be greatest when cocaine and alcohol are taken together. This is supported by several lines of evidence. For example, coadministration of ethanol with cocaine accelerates cocaine absorption, increasing the concentration of cocaine in brain extracellular fluid [19]. Ethanol also enhances cocaine-induced increases of extracellular DA in the nucleus accumbens [20], a critical brain area in the reward pathway [21]. Finally, preclinical self-administration and conditioned place preference (CPP) results, and clinical findings in humans, suggest that the rewarding properties of ethanol combined with cocaine are greater than those of each drug alone [22]. Similar findings are reported across species, including planarians [23], rodents [24, 25], non-human primates [26, 27], and humans [17, 28].

Cocaine and ethanol each increase extracellular levels of DA, NE, and 5-HT, neurotransmitters linked to drug reward. Cocaine does this by blocking the high-affinity transporters for these neurotransmitters: the DA transporter (DAT), NE transporter (NET), and 5-HT transporter (SERT), respectively [29]. While alcohol has a broad and complex range of actions [30], which includes increasing extracellular levels of monoamines [31], how it does this remains unclear.

Alcohol inhibits monoamine uptake (e.g., [32–36]), but we and others provide evidence that ethanol lacks activity at DAT [33], NET [34], and SERT [36]. Instead, organic cation transporter 3 (OCT3) could potentially contribute to ethanol’s monoamine uptake inhibition. OCT3 is a low-affinity transporter capable of transporting DA, 5-HT, and NE at high capacity (also known as an “uptake-2” transporter) [37, 38]. OCT3, located on neurons and glia in reward-related brain regions [39–44], may be an important regulator of monoamines [39–41, 43, 45–54]. Supporting a role for OCT3 in effects of ethanol, we found that ethanol-induced inhibition of 5-HT clearance from hippocampus was greater in SERT knockout (KO, -/-) mice than in wild-type (+/+) mice [36], corresponding with greater OCT3 expression in SERT-/- than SERT+/+ mice [54, 55]. We also found that histamine (a substrate for OCT3 [48]) is cleared faster from hippocampal extracellular fluid in SERT-/- mice than SERT+/+ mice [54]. Together, these findings suggest that activity of ethanol at OCT3 may account for greater inhibition of 5-HT clearance in SERT-/- mice than in SERT+/+ mice [36]. More support for ethanol’s action at OCT3 comes from findings that ethanol inhibits uptake of the prototypical cation, tritiated 1-methyl-4-phenylpyridinium ([^3^H]MPP^+^), into human epithelial colorectal adenocarcinoma (Caco-2) cells, which are rich in OCT3 [56].

Literature evidence supports the premise that ethanol inhibits DA, NE, and 5-HT clearance from extracellular fluid but does so in a manner independent of high-affinity, “uptake-1” transporters, DAT, NET, and SERT [33, 34, 36]. This raises the possibility that ethanol’s actions on monoamine uptake are mediated, at least in part, via OCT3, and provides a potential mechanism by which ethanol (via inhibition of OCT3) enhances cocaine-induced increases in extracellular monoamines, as well as cocaine’s rewarding properties. Here we focus on DA because of its important role in reward pathways [57] and provide novel evidence that ethanol acts in an OCT3-dependent manner to inhibit DA clearance and augment the rewarding properties of cocaine.

## Materials and methods

Naïve adult male and female OCT3+/+ and OCT3-/- mice bred on a C57BL/6 background were used for in vivo chronoamperometry, conditioned place preference (CPP) and locomotor sensitization studies. Male OCT3+/+, OCT3-/-, SERT+/+ and SERT-/- mice were used for loss of righting reflex (LORR) studies. All mice were from our in-house colonies. For details, see Supplementary Information (SI).

To assess binding of ethanol to the orthosteric site of DAT, NET and/or SERT we measured its ability to displace binding of [^3^H]WIN35,428, [^3^H]nisoxetine and [^3^H]citalopram, respectively, from striatal (DAT) or hippocampal (NET, SERT) homogenates, compared with high-affinity ligands for DAT, NET and SERT (GBR12909, reboxetine and citalopram, respectively) using established methods [58–60] (and SI).

To gain direct insight into ethanol’s interference with OCT3-mediated uptake we measured inhibition of [^3^H]MPP^+^ (Perkin Elmer, Boston, MA, USA) uptake into human embryonic kidney (HEK293) cells stably expressing the human (h) isoform of OCT3 (hOCT3), or mock transfected HEK293 cells, according to Janowsky et al. [61]. Additionally, we used HEK293 cells stably expressing the yellow fluorescent protein (YFP) tagged (to the N-terminus) isoforms of either hOCT3, hDAT, hNET or hSERT, and measured the ability of ethanol, cocaine (DAT, NET, SERT blocker) or corticosterone (OCT3 blocker) to inhibit uptake of the fluorescent substrates 4-(4-diemethylamino-styryl)-N-methylpyridinium (ASP^+^ for OCT3) or 4-(4-(dimethylamino)phenyl)-1-methylpyridinium, (APP^+^ for DAT, NET and SERT) according to established methods [44, 62, 63] (see SI).

High-speed chronoamperometry was used in vivo to assess the ability of ethanol to inhibit DA clearance, and to enhance the ability of cocaine to do so, in dorsal striatum according to established methods where a Nafion-coated carbon fiber electrode is coupled to a glass multibarreled micropipette containing DA and drugs of interest [50, 64, 65, 66] (see SI). Barrels were filled with either dopamine (200 μM), ethanol (100 mM), cocaine (400 μM), ethanol + cocaine, or vehicle (phosphate-buffered saline, (PBS)). Pressure-ejection of ~20 nL of 200 µM dopamine, 200 µm away from the recording electrode, results in signal amplitudes of ~0.5–1.0 µM [65], consistent with those elicited by stimulated release of DA [67]. Thus, the concentration of neurotransmitter and drug reaching the recording electrode is ~200–400 fold less than the barrel concentration. Consequently, ethanol’s concentration at the recording site is ~0.5–10 mM, consistent with extracellular brain concentrations after systemic administration of 1 g/kg of ethanol [68, 69]. Cocaine was locally applied to dorsal striatum to yield concentrations at the recording site of 1–10 μM, consistent with extracellular brain concentrations after systemic administration of behaviorally relevant doses of cocaine [70, 71]. Importantly, these concentrations of cocaine robustly inhibit DA clearance, whereas lower concentrations are known to increase DA clearance by trafficking DAT to the plasma membrane [72].

Rewarding effects of cocaine, ethanol, and their combination were measured by CPP using established methods [73, 74]. In the same procedure, sensitization of cocaine-induced locomotion (in the presence and absence of ethanol) was measured. Loss of righting reflex (LORR) was assessed as described previously [36]. See SI for details.

Competition binding and uptake of [^3^H]MPP^+^ into hOCT3 HEK293 cells were analyzed using non-linear regression, and means were compared by ANOVA. Uptake of APP^+^ and ASP^+^ in cells, chronoamperometry and behavioral data were analyzed by ANOVA and post-hoc analyses described in the figure legends. Assumptions underlying the statistical tests were met. Results are expressed as mean and standard error (SEM) or 95% confidence interval (CI). Tests were performed with GraphPad Prism (GraphPad, La Jolla, CA, USA), with statistical significance defined as *p* < 0.05. See SI for details.

## Results

### Ethanol does not displace radioligand binding to DAT, NET, and SERT in mouse brain tissue

**Fig. 1:**
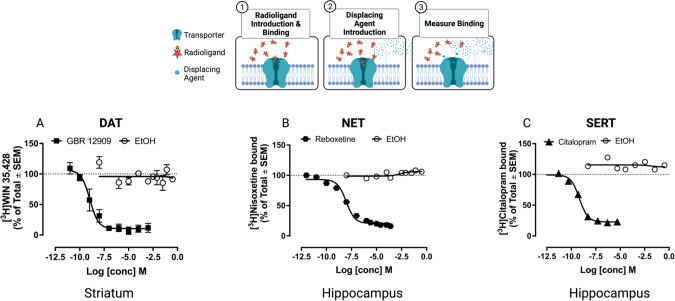
Ethanol fails to displace radioligand binding to DAT (A), NET (B) and SERT (C). In contrast, known inhibitors of these transporters displaced binding with Ki values in the nM range, as expected (see Table 1). Data shown are mean and SEM, *N* = 3 mice per assay. Where the error bar is not visible, it is obscured by the symbol. *Note*: Radioligand binding to **DAT** was performed on striatal homogenates using 2.7 nM [^3^H]WIN 35,428, unlabeled GBR 12909 at 11 concentrations from 0.01 nM to 1 mM and ethanol from 3.4 nM to 1.0 M. Radioligand binding to **NET** was performed on hippocampal homogenates using 1.7 nM [^3^H]nisoxetine, unlabeled reboxetine at 11 concentrations from 100 pM to 250 µM, and ethanol from 3.4 nM to 1.0 M. Radioligand binding to **SERT** was performed on hippocampal homogenates using 1.5 nM [^3^H]citalopram, unlabeled citalopram at 7 concentrations ranging from 5 pM to 5 µM and ethanol from 3.4 nM to 1.0 M. Cartoon created with Biorender.com.

Ki values for displacement of [^3^H]WIN35,428, [^3^H]nisoxetine, and [^3^H]citalopram binding to DAT, NET, and SERT by GBR12909, reboxetine, and citalopram, respectively, in striatal (for DAT) or hippocampal (for NET and SERT) homogenates were in the low nanomolar range, consistent with previous reports [58–60] (Fig. 1, Table 1). In contrast, ethanol (up to 1 M) did not displace [^3^H]WIN35,428, [^3^H]nisoxetine and [^3^H]citalopram, evidencing that ethanol does not interact with binding sites of well-established ligands at DAT, NET and SERT (Fig. 1, Table 1).

**Table 1:** Ki values for displacement of radioligands for DAT, NET and SERT by known inhibitors in mouse brain homogenates.

Transporter	Hot ligand	nM	Kd	Cold ligand	Ki (nM)
DAT	[^3^H]WIN35,428	2.7	7.8	GBR12909	1.00 (0.01 to 79)
NET	[^3^H]Nisoxetine	1.7	2.5	Reboxetine	1.26 (0.10 to 32)
SERT	[^3^H]Citalopram	1.5	1.5	Citalopram	0.32 (0.20 to 0.40)

Data are shown as mean (95% CI).

### Ethanol inhibits [^3^H]MPP^+^ uptake in hOCT3 HEK293 cells at concentrations relevant in vivo

**Fig. 2:**
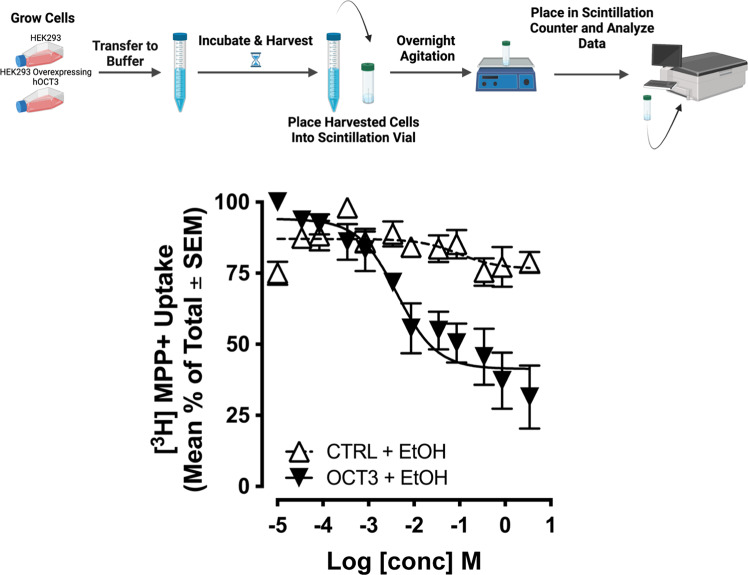
Ethanol inhibits [^3^H]MPP^+^ uptake in hOCT3 HEK293 cells to a greater extent than in mock-transfected cells. Assays were performed in triplicate. *N* = 5 replicate assays. As ethanol concentrations increased, inhibition of uptake was greater in hOCT3 expressing cells than in mock-transfected cells, *p* < 0.05. Filled symbols represent significantly different from mock-transfected cells. Data are shown as mean and SEM. Cartoon created using Biorender.com.

To directly assess putative activity of ethanol at OCT3, we used HEK293 cells stably expressing hOCT3 [75], examined ethanol’s inhibition of [^3^H]MPP^+^ uptake into these cells, and obtained a Ki = 4.2 ± 0.2 mM (Fig. 2). Ethanol inhibited uptake into hOCT3 transfected cells to a greater extent than into mock-transfected control cells (interaction *F*(11, 88) = 10.66, *p* < 0.0001; ethanol concentration *F*(11,88) = 17.3, *p* < 0.001; cell type *F*(1,8) = 10.18, *p* = 0.013) (Fig. 2). Because (i) systemic administration of 1 mg/kg of ethanol (i.p.) results in dialysate ethanol levels of approximately 6 mM in the nucleus accumbens of rats [68, 69], and (ii) human subjects may achieve similar [76] or considerably higher brain ethanol levels, with an emphasis on regular ethanol consumers [77], these results are consistent with ethanol inhibiting activity of OCT3 at behaviorally/physiologically relevant concentrations.

### Ethanol concentration-dependently inhibits uptake of fluorescent substrate via hOCT3 at concentrations that do not interfere with hDAT, hNET and hSERT-mediated uptake in vitro

**Fig. 3:**
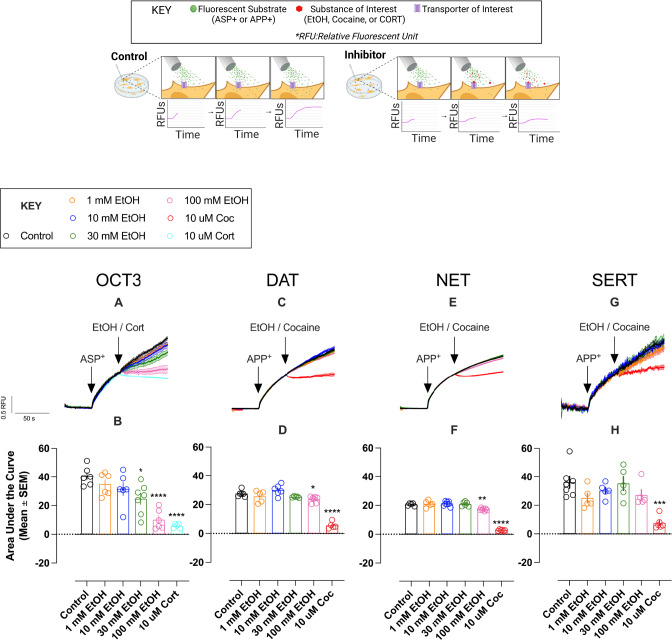
Real time assessment of substrate uptake reveals ethanol inhibits uptake at physiologically relevant concentrations in OCT3-expressing cells, but not DAT, NET or SERT expressing cells. **A** EtOH inhibited accumulation of ASP^+^ (3 µM) via OCT3 in a concentration-dependent manner. Addition of the OCT3-inhibitor corticosterone (cort, 10 µM) is shown as control. **B** Total A.U.C. (T_80-150 s_) from data shown in **A**. **C**, **E** Ethanol was without effect on DAT- or NET-mediated uptake at physiologically relevant concentrations and was without effect on SERT-mediated uptake (**G**). Addition of cocaine (10 µM) inhibited intracellular accumulation of APP^+^ (10 µM) via DAT, NET, and SERT (**C**, **E**, **G**). **D**, **F**, **H** Total A.U.C. (T_80-150 s_) from data shown in **C**, **E**, **G**. Data are shown as mean and SEM of 5-8 individual recordings per condition. Data shown in **B**, **D**, **F**, & **H** were analyzed using Brown-Forsythe ANOVA test, Dunnett’s T3 multiple comparisons test versus control. *p < 0.05, **p < 0.01, ***p < 0.001, ****p < 0.0001. Cartoon created with Biorender.com.

To further probe activity of ethanol at OCT3 versus “uptake-1” [37, 78] monoamine transporters, we measured its ability to inhibit uptake of fluorescent substrates in HEK293 cells expressing either the YFP tagged human isoforms of OCT3, DAT, NET or SERT. Uptake of the fluorescent compounds APP^+^ and ASP^+^ afford the temporal resolution to monitor instantaneous effects of test drugs on transporter activity, providing a direct readout of substrate transport that is mediated by electrochemical gradient-driven OCT3 and the secondary-active “uptake-1” transporters. Cells were superfused with fluorescent substrate (10 µM of APP^+^ for SERT, DAT and NET; 3 µM of ASP^+^ for OCT3) for 40 s to establish the initial uptake rate before cells were exposed to ethanol or well-established inhibitors of these transporters. As in previous studies, we used ASP^+^ to measure OCT3-mediated transport fluxes [44, 63]. Owing to the antagonistic properties of ASP^+^ at SERT [79], APP^+^ was used for SLC6 transporters, as it reliably labels DAT- and NET-expressing cells and behaves as a substrate of SERT [79, 80]. Ethanol concentration-dependently inhibited substrate uptake only in hOCT3 HEK293 cells (*F*(5, 22.62) = 16.99, *p* < 0.0001; Fig. 3A, B). Both the selective OCT3 blocker corticosterone (10 µM; *t*(5.16) = 12.37, *CI*_*95*_: 24.81, 44.72, *p* < 0.0001) and ethanol (100 mM; *t*(9.99) = 7.78, *CI*_*95*_: 18.28, 42.55, *p* < 0.0001), fully blocked uptake of ASP^+^, while 30 mM ethanol inhibited uptake by ~50% (*t*(10.29) = 3.31, *CI*_*95*_: 0.99, 30.74, *p* = 0.04). In contrast, ethanol did not impact fluorescent substrate uptake in hSERT HEK293 cells (Fig. 3G, H), and only modestly inhibited uptake into hDAT (*F*(5, 16.58) = 53.95, *p* < 0.0001; Fig. 3C, D) and hNET (*F*(5, 15.52) = 162.40, *p* < 0.0001; Fig. 3E, F) HEK293 cells at the highest concentration tested (100 mM). As expected, the DAT, NET, SERT blocker cocaine (10 µM) blocked APP^+^ uptake into cells expressing these transporters (Fig. 3C–H). We have previously shown that cocaine does not block fluorescent substrate uptake in hOCT3 HEK293 cells [44], which further supports the interpretation that the combination affects transporters of multiple families, i.e., SLC22 (OCT3 [37]) and SLC6 (DAT, NET, and SERT [81]).

### Ethanol inhibits DA clearance in dorsal striatum, and enhances cocaine-induced inhibition of DA clearance, in an OCT3-dependent manner

**Fig. 4:**
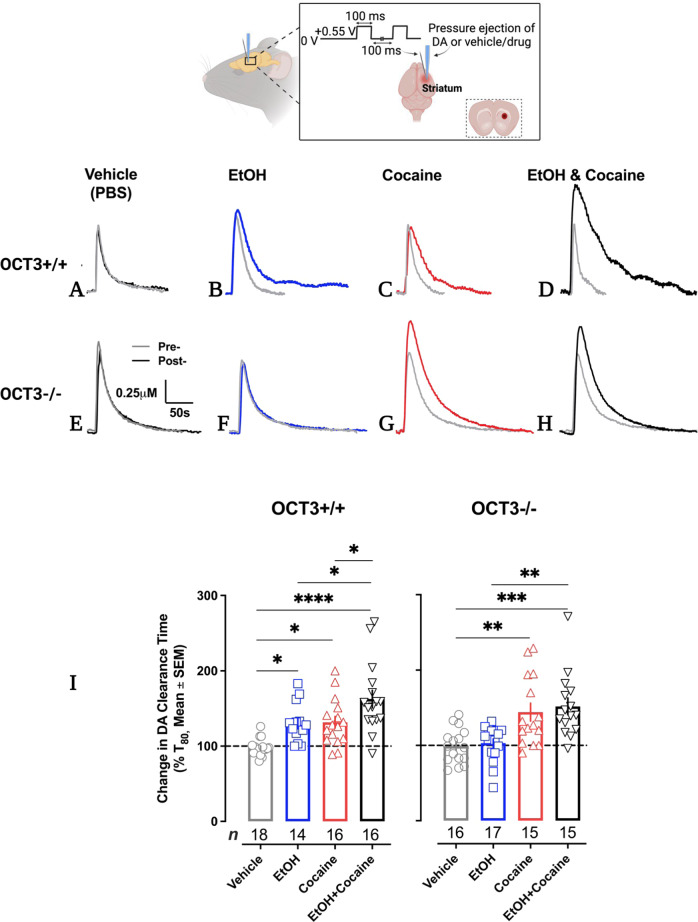
Ethanol inhibits DA clearance in dorsal striatum, and enhances cocaine-induced inhibition of DA clearance, in an OCT3-dependent manner. Representative traces of oxidation currents (converted to a micromolar value using a calibration factor determined in vitro prior to using electrodes in vivo) showing that in OCT3+/+ mice, vehicle had no effect on DA clearance time (T_80_) (**A**), whereas ethanol (~0.5-10 mM, see methods) (**B**) and cocaine (1–10 µM, see methods) (**C**) inhibited DA clearance. Coadministration of ethanol and cocaine inhibited DA clearance to a markedly greater extent than either drug alone (**D**). As expected, in OCT3-/- mice, vehicle had no effect on DA clearance (**E**), and cocaine inhibited DA clearance (**G**). However, unlike in OCT3+/+ mice, ethanol did not inhibit DA clearance (**F**), nor did it enhance the ability of cocaine to inhibit DA clearance (**H**). Summary data are shown in (**I**). Data are shown as mean and SEM change in T_80_ value 2 minutes following administration of drug or vehicle, *n* = 14–18 per condition. Data were analyzed using thee-factor ANOVA and followed up with separate 1-way ANOVAs per genotype, with Tukey’s test for post-hoc multiple comparisons. *p < 0.05, **p < 0.01, ***p < 0.001, ****p < 0.0001. Cartoon created with Biorender.com.

Important for the translational relevance of our in vitro data, which strongly point to OCT3 as a mechanism by which ethanol inhibits monoamine uptake, we used high-speed chronoamperometry to investigate effects of ethanol on OCT3-mediated DA clearance in vivo. We measured clearance of exogenously applied DA (to achieve signal amplitudes of ~0.5–1.0 μM) to dorsal striatum, a brain region implicated in the locomotor stimulant and rewarding effects of cocaine, in OCT3+/+ and OCT3-/- mice and tested the effect of ethanol (0.5–10 mM) and cocaine (1–10 µM), also locally applied to dorsal striatum, to inhibit DA clearance (see methods and SI methods for details). There were no statistically significant main or interaction effects of sex for any of the dependent variables, so data from males and females were pooled. Dependent variables were peak amplitude of the DA signal, time to clear between 20% and 60% of the peak signal (T_20-60_, the pseudo linear portion of the descending limb of the DA signal), and the time to clear 80% of peak signal (T_80_). The most marked effects were on T_80_. There were significant differences in DA clearance time (T_80_) among groups administered ethanol, cocaine, or ethanol and cocaine for OCT3+/+ (*F*(3, 60) = 12.36, *p* < 0.0001 (Fig. 4)) and OCT3-/- (*F* (3, 59) = 9.52, *p* < 0.0001 (Fig. 4)) mice. Intrastriatally applied ethanol increased T_80_ DA clearance time (i.e., inhibited DA clearance) in OCT3+/+ animals (*p* = 0.01), an effect that was lost in OCT3-/- mice (*p* = 0.98), consistent with OCT3 being a key player in the action of ethanol to inhibit DA uptake. As expected, a maximally effective pmol amount of cocaine locally applied to dorsal striatum inhibited DA clearance in both OCT3+/+ (*p* = 0.01) and OCT3-/- (*p* = 0.003) mice (Fig. 4). Examining different pmol amounts of cocaine confirmed that there were no significant differences in the maximal effect, or EC_50_ value, of cocaine to inhibit DA clearance between OCT3+/+ and OCT3-/- mice (see SI Fig. 1). Consistent with ethanol and cocaine acting at different sites, ethanol enhanced cocaine’s inhibition of DA clearance in OCT3+/+ mice (*p* = 0.03), but not in OCT3-/- animals (*p* = 0.93) (Fig. 4). Drug effects on T_20-60_ clearance time followed similar patterns (SI Table 1). Drugs generally did not have significant effects on signal amplitude. Although cocaine trended to increase signal amplitude in OCT3-/- mice, the only significant effect on amplitude was in OCT3+/+ mice following ethanol and cocaine, which increased DA signal amplitude (SI Table 2).

There were significant differences between genotypes for baseline (i.e., pre-drug) T_80_ values. DA clearance time was significantly longer in OCT3-/- mice (T_80_ 49 ± 3 s, *n* = 63) than their OCT3+/+ counterparts (T_80_ 37 ± 2 s, *n* = 64) (T_80_ *t*_125_ = 2.72, *p* = 0.01). Likewise T_20-60_ values were longer in OCT3-/- mice than OCT3+/+ mice (see SI). Baseline signal amplitude was not significantly different between genotypes, which was expected given we controlled the amount of DA pressure-ejected to yield similar signal amplitudes between genotypes. Neither the volume or pmol amount of DA delivered differed as a function of genotype (see SI).

### Reinforcing and locomotor sensitizing effects of cocaine are enhanced by OCT3-dependent actions of ethanol

**Fig. 5:**
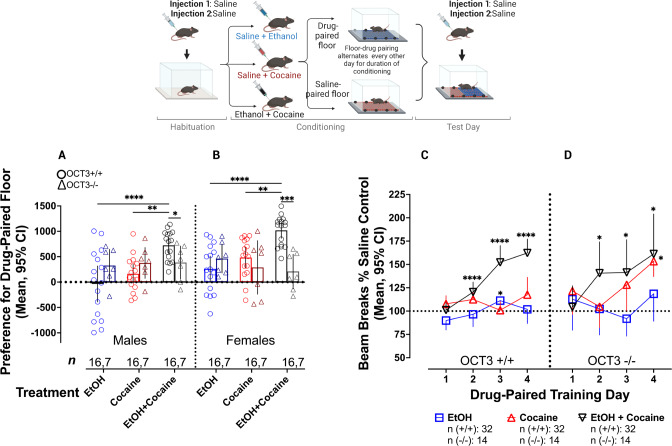
Ethanol enhances the rewarding and locomotor sensitizing effects of cocaine in an OCT3-dependent manner. **A**, **B** Ethanol (1 g/kg) enhanced CPP for cocaine (3.2 mg/kg) in male and female OCT3+/+ mice. This effect was lost in OCT3-/- mice. **C** In OCT3+/+ mice, the combination of cocaine and ethanol resulted in sensitization to locomotor stimulant effects of this drug pairing, whereas, at the doses used, neither drug alone produced sensitization. **D** OCT3-/- mice developed sensitization to cocaine, but coadministration with ethanol did not significantly enhance this effect at drug paired training days 3 and 4. *N* = 7-16/condition. Error bars represent 95% CI. Post-hoc comparisons for CPP were conducted with Tukey’s post-hoc, whereas Dunnet’s post-hoc test versus day 1 was used for sensitization analysis. *p < 0.05, **p < 0.01, ***p < 0.001, ****p < 0.0001. Cartoon created with Biorender.com.

To determine whether the ability of ethanol to augment cocaine-induced inhibition of DA clearance mapped onto behavioral outcomes in an OCT3-dependent manner, we assessed CPP and sensitization to the locomotor stimulant effects of cocaine in the presence and absence of ethanol. The CPP protocol is often used to model abuse-related drug effects, and is demonstrated by an animal’s preference for an environment that has been previously paired with a drug of interest in comparison to an environment paired with vehicle [82, 83]. Based on pilot studies (data not shown), we selected a dose of cocaine (3.2 mg/kg) and ethanol (1 g/kg) that generally failed to produce CPP, and in cases where CPP developed (e.g., female OCT3+/+ mice given cocaine), it was only modest (see SI methods). Three-way ANOVA was used to assess CPP in OCT3+/+ and OCT3-/- animals receiving ethanol, cocaine, or ethanol+cocaine (see Fig. 5).

We found significant interactions between treatment and genotype (*F*(2,126) = 10.68, *p* < 0.0001) as well as genotype and sex (*F*(1,126) = 5.02, *p* = 0.03). There was also a main effect for treatment (*F* (2, 126) = 6.74, *p* = 0.002). Consistent with abundant literature [84–89], cocaine elicited significant CPP in female OCT3+/+ animals (*p* = 0.002) (Fig. 5B). Also in line with previous literature [90–93], in wild-type mice co-administration of cocaine and ethanol led to a significant increase in preference for the drug-paired floor compared to either drug administered alone for both males (ethanol vs. ethanol+cocaine: *p* < 0.0001; cocaine vs. ethanol+cocaine: *p* = 0.001, Fig. 5A) and females (ethanol vs. ethanol+cocaine: *p* < 0.0001; cocaine vs. ethanol+cocaine: *p* = 0.002, Fig. 5B). Moreover, OCT3 +/+ male and female mice displayed a greater preference for the ethanol+cocaine-paired floor compared to male (treatment*genotype: *F*(2,63) = 3.54, *p* = 0.035, Fig. 5A) and female (treatment*genotype: *F*(2,63) = 7.83, *p* = 0.001, Fig. 5B) OCT3-/- mice. There was no significant effect of treatment on drug-paired floor preference for male or female OCT3-/- mice (Fig. 5A, B). Although the sample size for OCT3-/- mice was approximately half that for OCT3+/+ mice, this smaller size still afforded adequate power (i.e., 0.97, calculated with G*Power) to detect CPP differences like those observed in OCT3+/+ mice (i.e., Cohen’s *d* = 1.5-1.6 using Student’s t-test to compare the combination of ethanol and cocaine with either drug alone). Because the sample size in OCT3-/- mice afforded more than 80% power to detect CPP differences like those observed in OCT3+/+ mice, we truncated our CPP studies in OCT3-/- mice for ethical reasons.

Sensitization describes the phenomenon of enhanced locomotor-stimulating effects that are observed in response to repeated drug administration, thought to reflect brain changes leading to drug addiction [88]. Importantly, it appears sensitization is involved only in certain phases of drug addiction, and is thought to be an important initial step in the drug addiction process [94]. Sensitization to the locomotor stimulant effects of cocaine was first assessed using a four-way repeated measures ANOVA with Greenhaus-Geisser correction, with sex, genotype, treatment, and training day as independent variables. There were no interaction or main effects of sex on sensitization, so male and female data were pooled for further analysis. There was a significant three-way interaction of drug-paired training day, treatment, and genotype (*F* (5.282, 348.59) = 4.02, *p* = 0.001). To examine this three-way interaction in more detail, the interaction between drug-paired training day and genotype was assessed separately for each treatment condition. There was no effect of training day or genotype on the effect of ethanol to impact locomotor activity (Fig. 5C, D). There was no ethanol-induced sensitization. For the cocaine-treated group, there were significant main effects of training day (*F*(2.63, 115.8) = 3.82, *p* = 0.02) and genotype (*F*(1, 44) = 8.84, *p* = 0.01). Interestingly, OCT3-/- (first vs. 4th session: Mean Difference = -31.91, *p* = 0.04, Fig. 5D), but not OCT3+/+ (first vs. 4th session: Mean Difference = -9.84, *p* = 0.75, Fig. 5C) mice developed sensitization to the locomotor stimulant effects of cocaine alone, suggesting that OCT3-/- mice are more sensitive to this effect of cocaine than their wild-type counterparts. For the groups administered ethanol + cocaine, we observed a significant main effect of training day (*F* (2.67, 117.6) = 22.57, *p* < 0.0001), but no effect of genotype. OCT3+/+ mice administered both ethanol and cocaine developed significant sensitization to the locomotor stimulant effect of this drug combination (first vs. 4th session: Mean Difference = −61.19, *p* < 0.0001, Fig. 5C). Moreover, the locomotor increase from repeated coadministration of ethanol and cocaine was substantially greater compared to OCT3+/+ mice that received either ethanol (first vs. 4th session: Mean Difference = −12.01, *p* = 0.42) or cocaine (first vs. 4th session: Mean Difference = -9.84, *p* = 0.69) alone, (Fig. 5C), consistent with this drug combination amplifying the effect of either drug alone. OCT3-/- mice also developed sensitization to the locomotor stimulant effects of ethanol+cocaine (first vs. 4th session: Mean Difference = -56.92, *p* = 0.02, Fig. 5D). Importantly, consistent with activity of ethanol at OCT3, when given in combination with cocaine there was no further significant enhancement of sensitization to the locomotor stimulant effect of cocaine in OCT3-/- mice at days 3 and 4, though it appears the combination of drugs may have accelerated sensitization to cocaine, being evident at day 2 in OCT3-/- mice, whereas sensitization to cocaine alone was not apparent until day 3. This could indicate actions of ethanol elsewhere, but does not preclude a major role for OCT3 in the actions of ethanol.

### Lack of OCT3 Significantly Reduces Sensitivity to the Sedative/Hypnotic Effects of Ethanol

**Fig. 6:**
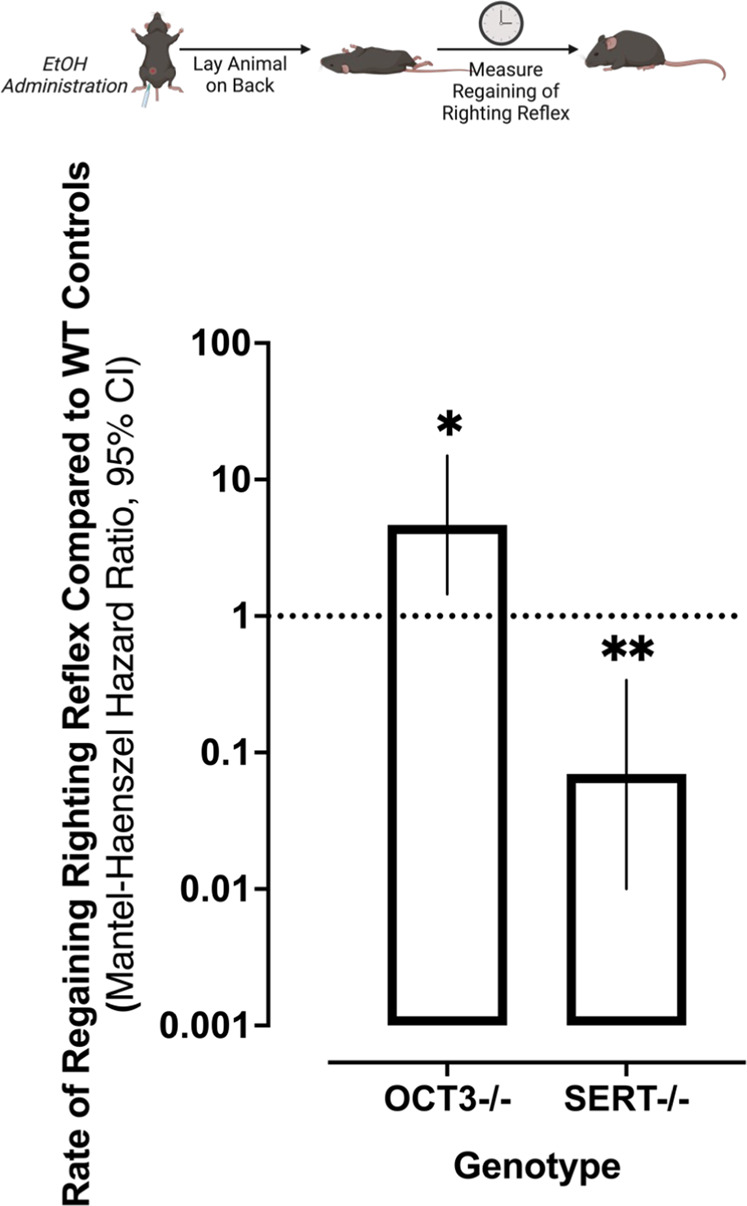
Sedative/hypnotic effects of ethanol are dampened in OCT3-/- mice and enhanced in SERT-/- mice. Replicating our previously published findings [36, 98] SERT-/- mice, which have greater expression of OCT3 than wild-type mice, administered ethanol (3.2 g/kg) regained their righting reflex at a significantly slower rate than their wild-type controls. In contrast, OCT3-/- mice regained their righting reflex significantly faster than wild-type controls. Data were analyzed via survival analysis, using the Mantel-Cox log-rank test and the Mentel-Haenszel hazard ratio followed by Holmes-Šidák correction for multiple comparisons. *p < 0.05, **p < 0.01 compared to within-genotype control mice; error bars represent 95% CI, *n* = 6 – 9. Cartoon created with Biorender.com.

Greater sensitivity to the sedative/hypnotic effects of ethanol is associated with a higher incidence of alcohol use disorder in humans [95]. We therefore investigated the ability of ethanol (3.2 g/kg) to induce loss of righting reflex (LORR), a commonly used indicator of sedative/hypnotic effects in rodents, in wild-type (OCT3+/+ and SERT+/+), OCT3-/- and SERT-/- mice. We included SERT-/- mice as a comparator because we have previously shown that they have higher expression of OCT3 than SERT+/+ mice, which is associated with longer LORR [36, 96], though the mechanism for this effect remains unknown. Replicating our previous findings [36, 96], SERT-/- mice regained their righting reflex at a rate 14.34 times slower than SERT+/+ controls (*χ*^*2*^(1) *=* 9.99, *p* = 0.003, *HR* = 14.34, *CI*_*95*_: 2.75, 74.78) indicating that these animals are significantly more sensitive to the sedative/hypnotic effects of alcohol (Fig. 6). Consistent with increased expression of OCT3 playing a mechanistic role in this finding, OCT3-/- mice regained their righting reflex at a rate approximately 4.65 times faster than OCT3+/+ controls (*χ*^*2*^(1)*=*6.64, *p* = 0.01, HR = 4.65, *CI*_*95*_: 1.45, 14.96), indicating that mice lacking OCT3 are significantly less sensitive to the sedative/hypnotic effects of ethanol, and that the sedative effects of ethanol appear to be dependent on degree of OCT3 expression. There was no effect of genotype on LORR latency (*χ*^*2*^(3) = 0.87, *p* = 0.83; data not shown).

## Discussion

Here we report the novel finding that ethanol acts at OCT3 to inhibit monoamine uptake. We base this on our findings that (1) ethanol inhibits fluorescent and radiolabeled substrate uptake in hOCT3 HEK293 cells at physiologically relevant concentrations; (2) in wild-type, but not in OCT3-/- mice, ethanol (a) inhibits DA clearance in striatum in vivo; (b) enhances the ability of cocaine to inhibit DA clearance in striatum in vivo; (c) augments CPP for cocaine; and (d) augments sensitization to the locomotor stimulant effects of cocaine; (3) sedative/hypnotic effects of ethanol are less in OCT3-/- mice than wild-type mice; and (4) ethanol does not displace binding of well-established ligands that bind to the S1 site of DAT, NET or SERT nor does it interfere with uptake of substrates via each transporter at concentrations lower than 100 mM. Together with our published findings that cocaine does not interact with OCT3 at physiologically relevant concentrations (e.g., concentrations that produce maximal behavioral effects) [44] these findings provide a novel mechanistic basis for the increased and prolonged subjective euphoria experienced by individuals who use cocaine and alcohol concurrently. By acting at different sites to inhibit monoamine uptake (i.e., ethanol at OCT3, cocaine at DAT, NET, and SERT), the ensuing increase in monoamines, and therefore conceivably the “high”, is greater when ethanol and cocaine are taken concurrently than when they are taken separately.

We focus our discussion on DA, since it is considered the primary player in rewarding effects of ethanol, however, since OCT3 also transports other cations with varying efficiencies [38, 97], our discussion applies to other monoamines as well. Among its many actions, ethanol increases extracellular DA, assessed predominanty using microdialysis, but the mechanisms by which it does are not clear. Often the increase in extracellular DA has been attributed to ethanol increasing DA release. However, this does not take into account that changes in microdialysate DA reflect a number of processes occurring in tandem, including DA release, uptake and metabolism. Electrophysiological studies show that ethanol increases firing of DA neurons [98–101], and there is some evidence that ethanol influences DA metabolism [102], whereas studies of the effect of ethanol on DA uptake have been mixed. This is likely due to different preparations and approaches used; however, using fast-scan cyclic voltammetry to measure DA clearance in striatum in vivo, Wightman’s group found that ethanol slowed DA clearance [32]. Subsequent studies from the Jones’ group, using striatal slices prepared from DAT-/- mice, showed this effect of ethanol to be DAT-independent [33]. These findings, together with those showing that ethanol potentiates cocaine-induced increases in extracellular DA [20] suggest that ethanol acts at a site(s) other than DAT to exert this effect. Our findings provide evidence that ethanol acts via OCT3 to inhibit DA uptake.

Because DAT, NET and SERT take up substrates other than their native neurotransmitters [37], we first investigated if ethanol had activity at these high-affinity “uptake-1” transporters to inhibit monoamine clearance. Evidence has been reported both for [32, 35, 103] and against [33, 34, 36] activity of ethanol at these transporters, with different observations likely influenced by use of different expression systems [104] and different concentrations of ethanol. We found that ethanol, at concentrations as high as 1 M, did not compete against, [^3^H]WIN35,428 binding to DAT in striatal homogenates, or against [^3^H]nisoxetine and [^3^H]citalopram binding to NET and SERT, respectively, in hippocampal homogenates. In contrast, high affinity ligands for these transporters (GBR12909, reboxetine and citalopram,) displaced [^3^H]WIN35,428, [^3^H]nisoxetine and [^3^H]citalopram to DAT, NET and SERT, respectively, with Ki values in the nM range. However, the pharmacology of monoamine transporters is complex and modulators may bind to ortho- and allosteric sites [105]. Ethanol interacts with a binding site that is located on the extracellular loop 2 of the DAT [106]. Hence, ligand-displacement assays carried out at equilibrium may not allow for identification of modulatory properties, or lack thereof. Consequently, we also performed functional assays to determine the effect of ethanol on DAT, NET, SERT and OCT3 with high temporal resolution. Ethanol at concentrations as high as 100 mM did not inhibit uptake of fluorescent substrate into hSERT HEK293 cells, and at this high concentration, only modestly inhibited uptake into hDAT and hNET HEK293 cells. In contrast, cocaine (10 µM) fully blocked substrate uptake in these cell lines. Combined with existing literature [33, 34, 36], our data support findings that ethanol does not perturb function of DAT, NET or SERT, with regards to inwardly-directed transport fluxes.

Rapidly growing evidence supports an important role for OCT3 in monoamine neurotransmission [39–41, 43, 45–54]. Because ethanol concentration-dependently inhibits uptake of [^3^H]MPP^+^ into Caco-2 cells, which are rich in OCT3 [56], we hypothesized that ethanol acts to inhibit monoamine uptake via actions at OCT3. Consistent with our hypothesis, we found that ethanol inhibited uptake of [^3^H]MPP^+^ into hOCT3 HEK293 cells with a Ki of 4.2 ± 0.2 mM. Considering that brain concentrations of ethanol reported for rodents and human subjects are in the low mM range [68, 76], this observation suggests that the inhibitory properties of ethanol at OCT3 contribute to elevations in extracellular monoamines observed following ethanol exposure. Consistent with this, ethanol inhibited fluorescent substrate uptake into hOCT3 HEK293 cells. These novel findings further support the conclusion that ethanol acts to inhibit monoamine uptake via its action at OCT3. Based on the prolonged exposure times (minutes) to ethanol during binding experiments and uptake utilizing [^3^H]MPP^+^ as a substrate, it remains possible that the antagonistic activity of ethanol at OCT3 involves other effects, such as disruptions of membrane composition and intracellular signaling cascades affecting OCT3 more than members of the SLC6 family. However, the temporal resolution of the fluorescent tracer-based uptake experiments allowed us to uncover an instantaneous effect of ethanol on OCT3-mediated transport processes. This observation strongly supports the interpretation that ethanol directly and instantaneously perturbs the function of OCT3.

To interrogate the translational relevance of these in vitro findings we turned to constitutive OCT3-/- mice. Consistent with earlier reports [32], ethanol inhibited DA clearance in striatum of wild-type (OCT3+/+) mice. This effect was lost in OCT3-/- mice, consistent with a crucial role for OCT3 in the actions of ethanol to inhibit DA clearance. We [44], and others [39] have previously shown that cocaine does not have activity at OCT3 at concentrations lower than 100 µM, and our data show that ethanol does not interfere with the function of DAT, NET or SERT. Consistent with these drugs acting at different sites, ethanol enhanced the ability of a maximally effective concentration of cocaine to inhibit DA clearance in striatum of OCT3+/+ mice, but not in OCT3-/- mice. The genotype-dependency of ethanol’s effects on DA clearance make non-specific effects of ethanol, such as fluidization of the cell membrane [107], unlikely. Related to our findings, the OCT3 blocker corticosterone has been shown to enhance cocaine-induced DA signaling and behaviors via nongenomic glucocorticoid actions, suggesting involvement of an OCT3-dependent mechanism [49, 52]. Taken together, these results suggest that cocaine-induced increases in DA signaling can be potentiated by concurrent blockade of OCT3. These findings are in line with our previous studies showing that the OCT blocker, decynium-22, enhances the antidepressant-like effects of SERT and NET blockers most likely via its action at OCT3 [50, 108].

Although preclinical investigations using self-administration and CPP across species [23, 25–28, 92], as well as clinical findings in humans [16–18], suggest that rewarding properties of the combination of ethanol and cocaine are greater than those of each drug alone, the mechanism(s) underlying these observations remain unknown. Consistent with these reports, ethanol potentiated CPP for cocaine in OCT3+/+ mice. This effect was more pronounced in females than males, consistent with findings that women are more sensitive to the effects of psychostimulants and progress from initial use to addiction faster than men [109, 110]. Importantly, in agreement with our in vivo neurochemical data, ethanol did not potentiate CPP for cocaine in OCT3-/- mice, revealing OCT3 as an important mechanism in this action of ethanol. Though no significant sex differences were observed in sensitization to the locomotor effects of ethanol, cocaine or their combination, doses of ethanol and cocaine that did not elicit sensitization when given alone, did when given together in OCT3+/+ mice. Interestingly, sensitization to the locomotor stimulant effect of cocaine did emerge in OCT3-/- mice, suggesting potential compensation in these mice as a result of constitutive ablation of OCT3. Importantly however, when cocaine was given together with ethanol in OCT3-/- mice, sensitization was not enhanced, again pointing to a crucial role for OCT3 in this effect of ethanol. In line with our observations, previous reports have shown that ethanol, at a dose that does not cause CPP per se, enhances the conditioning properties of the DAT, NET and SERT-targeting cathinone mephedrone [111] and enhances its effects on extracellular monoamines [112]. Related to potential compensation in constitutive OCT3 KO mice, available data suggest that there is no overt compensation that would account for the present findings [41, 44, 113] (see SI).

To examine the generality of our findings, we assessed the sedative/hypnotic effects of ethanol in wild-type, OCT3-/- and SERT-/- mice. We, and others, have shown OCT3 expression to be greater in SERT-/- mice than in wild-type mice [54, 55], and that ethanol’s inhibition of 5-HT clearance in hippocampus of SERT-/- mice is more robust than in SERT+/+ mice [36], consistent with a role of OCT3. Likewise, we found that the sedative/hypnotic effects of ethanol were more pronounced in SERT-/- mice [36, 96]. We replicated this finding, and extended it by showing that ethanol-induced LORR is less in OCT3-/- mice, consistent with OCT3 contributing to ethanol-induced LORR. Although we did not measure blood alcohol concentration in OCT3+/+ and OCT3-/- mice, differences in ethanol metabolism between the two genotypes are unlikely [36, 96] (see SI). Taken together with data presented here, it appears that LORR is related to level of OCT3 expression.

Collectively, our data provide compelling evidence for OCT3 as a novel and previously unsuspected player in the actions of ethanol to inhibit monoamine uptake. Here we identified OCT3 as an important mediator of the acute effects of ethanol to inhibit DA uptake and produce rewarding effects. These novel findings raise several avenues for exciting future studies, including investigations of (and SI for details) (1) the effect of chronic cocaine and ethanol use in combination or alone, (2) potential actions of cocaethylene, a psychoactive substance formed in the liver when cocaine and alcohol coexist in blood [114], at OCT3, as well as acetaldehyde, the main metabolite of ethanol, which is readily self-administered in rodents and could play a role in the rewarding properties of ethanol [115], (3) self-administration of ethanol, cocaine and their combination, since behavioral and neurochemical effects of most drugs of abuse, including ethanol and cocaine, differ depending on whether they are administered contingently vs. non-contingently (e.g., [116–118]), (4) how ethanol interacts with OCT3 to inhibit substrate uptake. These efforts will build on the recently published structural basis for OCT3 inhibition [119].

In sum, these data raise the provocative idea that it may be possible to pharmacologically prevent ethanol’s actions at OCT3, and/or to enhance OCT3 transporter function, as a therapeutic approach to treating alcohol dependence, and alcohol and cocaine co-abuse [120], and open an avenue rich for future research.

### Supplementary information

Supplemental Material

## Data Availability

Data supporting these findings are available from the corresponding author upon reasonable request.
