# Supplementary material for: Ethanol inhibits dopamine uptake via organic cation transporter 3: Implications for ethanol and cocaine co-abuse
Source: Mol Psychiatry. 2023 Jun 13;28(7):2934–45. doi: 10.1038/s41380-023-02064-5 (PMC10615754; doi:10.1038/s41380-023-02064-5)
Supplement: Supplementary file 1 — Supplemental Material [file 41380_2023_2064_MOESM1_ESM.docx]

**Supplementary Information**

Ethanol inhibits dopamine uptake via organic cation transporter 3: Implications for ethanol and cocaine co-abuse

Clauss, N.J., Ph.D. ^†1^, Mayer, F.P., Ph.D. ^†2#^, Owens, W.A., M.S.**^1^**, Vitela, M., B.S.**^1^**, Clarke, K.M., B.S.A.**^1^**, Bowman, M.A., Ph.D.**^1^**, Horton, R.E., B.S.**^1^**, Gründemann, D., Ph.D. ^3^, Schmid, D., M.D.^2^, Holy, M.^2^, Gould, G.G., Ph.D.**^1^**, Koek, W., Ph.D.^4^ Sitte, H.H., M.D.^2,5^, & Daws, L.C., Ph.D.^1,6*^

^1^ Department of Cellular and Integrative Physiology, University of Texas Health Science Center at San Antonio, San Antonio, TX, 78229, USA

^2^ Center for Physiology and Pharmacology, Medical University of Vienna, 1090, Vienna, Austria.

^3^ Department of Pharmacology, Faculty of Medicine and University Hospital Cologne, University of Cologne, 50931, Cologne, Germany

^4^ Department of Cell Systems and Anatomy, University of Texas Health Science Center at San Antonio, San Antonio, TX, 78229, USA

^5^ Center for Addiction Research and Science, Medical University Vienna, Waehringerstrasse 13 A, 1090, Vienna, Austria.

^6^ Department of Pharmacology, University of Texas Health Science Center at San Antonio, San Antonio, TX, 78229, USA

^†^ Contributed equally as first authors

^#^ Present Address: Department of Neuroscience, Faculty of Health and Medical Sciences, University of Copenhagen, DK-2200 Copenhagen, Denmark.

* Correspondence: daws@uthscsa.edu (LCD)

**Materials and Methods**

Naïve adult male and female OCT3+/+ and OCT3-/- mice bred on a C57BL/6 background from our in-house colonies were used for *in vivo* chronoamperometry, conditioned place preference (CPP) and locomotor sensitization studies. Male OCT3+/+, OCT3-/-, SERT+/+ and SERT-/- mice from our in-house colonies were used for loss or righting reflex (LORR) studies. Genotype comparisons were made among littermates, bred by +/- intercross to produce all three genotypes within a litter. Mice are backcrossed (+/+ x -/- to yield +/-) every 6^th^ generation. Mice were between 3 and 9 months of age for all experiments. Mice were housed in plastic cages (29 cm × 18 cm × 13 cm) containing 7090 Teklad sani-chip bedding (Envigo, East Millstone, NJ) and maintained on a 12/12 hr light/dark cycle (lights on at 7:00 am) in a temperature-controlled (24 °C) vivarium. Mice were weaned at postnatal day 21 and housed with same sex littermates with no more than 5 mice per cage. Food (Teklad LM-485 mouse/rat sterilizable diet 7012 chow; Envigo, East Millstone, NJ) and water were provided *ad libitum*. All procedures were conducted in accordance with the National Institute of Health Guide for the Care and Use of Laboratory Animals (Institute of Laboratory Animal, Resources, Commission of Life Sciences, National Research Council, https://grants.nih.gov/grants/olaw/Guide-for-the-Care-and-use-of-laboratory-animals.pdf), and with the approval of the Institutional Animal Care and Use Committee, The University of Texas Health Science Center at San Antonio.

To achieve at least 80% power to detect pre-specified effects of interest, sample sizes were estimated (by G*Power) from the smallest effect sizes considered to be important based on our previous publications (e.g.^1-14^) or theoretical considerations. In general, different groups in an experiment are unlikely to have identical population variances.  To maximize the robustness of the statistical analyses to violations of homogeneity, equal group sizes were planned. Mice were randomly assigned to each experimental condition. To avoid litter effects, no more than one mouse per litter was used in the same specific experimental condition. Investigator blinding during the behavioral experiments did not take place, because their outcomes did not involve direct observation but were recorded automatically. Experiments were carried out at the same time each day. An priori exclusion criterion was used for in vivo high-speed chronoamperometry. Data were excluded if the electrode and/or micropipette malfunctioned, or if mice died during the recording session.  Data of three animals were excluded.  No data were excluded from the behavioral experiments.

**Competition Binding Assays at Monoamine Transporters in Mouse Brain.**

*Mice***:** OCT3 mRNA and protein expression was found to be elevated in SERT +/- mice^14^. Thus, in the current experiments, male constitutive SERT+/- mice weighing 25-30g were used, 3 per assay. Functional SERT is reduced by 50% in SERT+/- mice, but SERT binding properties remain consistent and there is no evidence of compensatory upregulation of NET or DAT^15^. Mice were euthanized by cervical dislocation without use of anesthesia to prevent anesthetic-induced attenuation of transporter binding sites. Whole hippocampi or striatum were harvested and placed in 30 ml of ice-cold buffer specific to each assay.

*Drugs:* All chemicals were obtained from Sigma-Aldrich (Burlington, MA, USA) unless otherwise noted. Drugs were dissolved either in buffer, or for those with poor solubility, at their highest concentration stock solution of 100% dimethyl sulfoxide.

*Tissue Collection and Preparation*: Mice were humanely euthanized by cervical dislocation and decapitated without anesthesia to collect fresh brain tissues without compromising transporter binding properties. Striatum from each mouse were placed in a round 50 ml polycarbonate tube (Nalge Nunc, Rochester, NY, USA) containing 30 mM sodium phosphate and 0.32 M sucrose for DAT binding as in Reith and Coffey^16^. Hippocampi from each mouse were placed in a 50 ml tube containing 50 mM Tris-HCl, 120 mM NaCl, 5 mM KCl, pH 7.4 at 4°C for NET binding, or pH 7.4 at 25°C for SERT binding. All tissues were homogenized at 26,000 RPM for 1 min with a Polytron tissue homogenizer (Brinkman Instruments, Westbury, NY, USA). Homogenates were centrifuged for 10 min at 36,000 x g at 4°C using a JA 25.50 rotor (Avanti J-E, Beckman Coulter, Indianapolis, IN, USA). The supernate was discarded, and the pellet re-suspended in 25 ml buffer on ice using a Potter Elvehjem 10 ml glass and teflon homogenizer. The homogenate was re-centrifuged for 10 min at 36,000 x g. These final pellets were re-suspended to obtain an approximate protein concentration of 1 mg/ml, determined subsequently with Bradford reagent (Sigma, St. Louis, MO) and colorimetric detection at 595 nm by a plate reader (Spectra Max 190, Molecular Devices, San Jose, CA, USA).

*Homogenate Competition Binding***:**

*Radioligand binding to DAT* was performed on striatal homogenates using 2.7 nM [^3^H]WIN 35,428 (Perkin Elmer, Boston, MA, USA) and following the procedure of Price et al.^17^. The final pellet was re-suspended in 30 mM sodium phosphate and 0.32 M sucrose pH 7.4 at 4°C, and binding assays were carried out in duplicate for 2 hours at 4°C. Each reaction tube contained 100 ml of brain homogenate, and had a total volume of 250 ml. Unlabeled GBR 12909 at 11 concentrations from 0.01 nM to 1 mM and ethanol from 3.4 nM to 1.0 M (200 proof, Decon Labs, King of Prussia, PA, USA) were used for [^3^H]WIN 35,428 displacement, and values were transformed to % of total binding, which was 1318 ± 348 fmol/mg protein.

*Radioligand binding to NET* was performed on hippocampal homogenates using 1.7 nM [^3^H]nisoxetine (Perkin Elmer, Boston, MA, USA) following the procedure of Tejani-Butt^18^. The final pellet was re-suspended in 50 mM Tris, 300 mM NaCl, 5 mM KCl, pH 7.4 at 4°C, and binding assays were carried out in duplicate for 4 hours at 4°C in 50 mM Tris, 300 mM NaCl, 5 mM KCl. Each reaction tube contained 100 ml of brain homogenate, and had a total volume of 250 ml. Unlabeled reboxetine at 11 concentrations from 100 pM to 250 µM, and ethanol from 3.4 nM to 1.0 M was used for [^3^H]nisoxetine displacement, and values were transformed to % of total binding, which was 102 ± 12 fmol/mg protein.

*Radioligand binding to SERT* was performed on hippocampal homogenates according to the procedure of D’Amato et al.^19^. Competition binding assays were carried out in duplicate for 1 hour in 50 mM Tris, 120 mM NaCl, 5 mM KCl buffer, pH 7.4 at 25°C containing 1.5 nM [^3^H]citalopram (Perkin Elmer, Boston, MA, USA). Each reaction tube contained 100 ml of brain homogenate, and had a total volume of 250 ml. Unlabeled citalopram at 7 concentrations ranging from 5 pM to 5 µM and ethanol from 3.4 nM to 1.0 M were used for [^3^H]citalopram displacement and values were transformed to % of total binding, which was 36 ± 2.8 fmol/mg protein.

*Termination of Assays and Labeled Tissue Collection***:** Incubation for [^3^H]WIN35,428, [^3^H]nisoxetine or [^3^H]citalopram assays was terminated by three additions of 4 ml of buffer, pH 7.4 at 4°C. Labeled homogenates were captured through filtration under vacuum onto glass fiber filters pre-soaked in 0.5% polyethyleneimine (Sigma) on a Brandel tissue harvester (Gaithersburg, MD). Filters were washed twice more with 4 ml of buffer. Radioactivity trapped by the filters was measured using Beckman 6500 (Beckman, Brea, CA) with efficiency of 40-65%.

*Data Analysis*: Binding data were analyzed using non-linear regression curve fitting with GraphPad Prism (La Jolla, CA, USA). To calculate Ki values with Prism, known Kd values from prior studies were used for citalopram 1.5 nM^6^, and nisoxetine 2.5 nM^20^. Saturation assays were performed on striatum of 5 C57BL/6J mice to obtain Kd = 7.8 ± 2 nM for [^3^H]WIN35,428.

**Ethanol Displacement of [^3^H]MPP^+^ in Human Embryonic Kidney 293 Cells Over-expressing human OCT3.**

*Cells*: To examine more specifically at what concentration ethanol may displace or disrupt OCT3 binding, we used tritium labeled 1-methyl-4-phenyl-1,2,3,6-tetrahydropyridine cation ([^3^H]MPP^+^, Perkin Elmer, Boston, MA, USA) in competitive inhibition uptake assays in human embryonic kidney (HEK) 293 cells as vector controls or HEK 293 cells over-expressing human (h) OCT3 (generated in the Gründemann lab). Assays were performed with [^3^H]MPP^+^ at the same time in hOCT3 over-expressing and vector cells to determine the Ki of ethanol. Uptake buffer consisting of 25 mM HEPES, 120 mM NaCL, 5 mM KCl, 2.5 mM CaCl_2_, and 1.2 mM MgSO_4_, 1μM pargyline, 2 mg/ml glucose and 0.2 mg/ml ascorbic acid, pH 7.4 exactly as described in Janowsky et al.^21^ was used for these assays. Mechanically detached HEK293 cells grown to confluence in 4-5 ml flasks were suspended in 3-4 ml of media in a 15 ml polypropylene conical tube. Cells had an average size of 5 μm, count between 9.8x10^5^ and 2.3x10^6^/ml when measured 16 hours later.

*Assay Termination and Data Collection***:** For all HEK 293 cell assays, incubations were terminated by partial submersion of tubes in an ice water bath and addition of 3 ml buffer to the tubes. Cells were harvested on a Brandel 48 well harvester by vacuum filtration onto glass fiber filter strips (Brandel, Gaithersburg, MD, USA) pre-soaked in a 1% polyethalineamine saline solution. Dried filter circles were placed in 6 ml plastic vials, agitated overnight on a low-speed orbital shaker, and placed in a scintillation counter (Beckman LS6500, Brea, CA, USA) and counted for [^3^H] for 5 min each at 68.2% efficiency. Protein concentration of the cell suspension was determined after solubilization in 1N NaOH against known concentrations of bovine serum albumen with Bradford reagent as for mouse brain homogenates.

*Data Analysis*: Experiments were performed in triplicate. Percent of total binding was calculated, and uptake kinetics determined with GraphPad Prism. Km = 1 mM for [^3^H]MPP^+^ found in the OCT3 over-expressing cells was used to determine Ki values. Specific [^3^H]MPP^+^ uptake, was greater than in mock-transfected cells, consistent with prior reports^5, 21-24^.

**Uptake of fluorescent substrates in hOCT3, hDAT, hNET and hSERT cell lines.**

*Cell culture:* HEK293 cells were grown in Dulbecco’s modified eagle’s medium (DMEM), supplemented with 10% heat-inactivated fetal calf serum, streptomycin (100 µg / 100 mL), and penicillin (100 U / 100 mL). Cells were maintained at a sub-confluential state at 37°C in a humidified atmosphere (5% CO_2_). HEK293 cells stably expressing either the yellow fluorescent protein (YFP) tagged human isoforms of OCT3, DAT, NET or SERT, (the tag was attached to the amino-terminus), generated in the Sitte lab, were plated on poly-D-lysine coated 3.5 cm CELLVIEW™ (Greiner Bio-one, Kremsmuenster, Austria) cell culture dishes 24 h prior to the experiment. Stable cell lines were established by transfecting the transporters of interest by using the CaPO_4_ method, and stable clones were by limited dilution^2, 8^. G-418 (50 µg/mL) was added to the cell culture medium to maintain selection pressure.

*Uptake assay:* Uptake of fluorescent substrates was recorded as described previously^2^. During the experiment, the cell culture dish was continuously superfused with Krebs-HEPES buffer (120 mM NaCl, 3 mM KCl, 2 mM CaCl_2_, 2 mM MgCl_2_, 20 mM d-glucose monohydrate, 10 mM HEPES, pH 7.3-7.4; 25°C) to avoid accumulation of non-specific fluorescence. Cells were superfused with fluorescent substrate (i.e. 10 µM of 4-(dimethylamino)phenyl)-1-methylpyridinium (APP^+^) for DAT, NET and SERT; 3 µM of 4-(4-(dimethylamino)styryl)-N-methylpyridinium (ASP^+^) for OCT3) for 40 seconds to establish the initial uptake rate in absence of inhibitors. Subsequently, the microsuperfusion system was switched to buffers containing fluorescent substrate and the substances of interest (ethanol, cocaine, corticosterone). Each trace was normalized to the relative fluorescent units immediately prior to the addition of ethanol, cocaine (10 µM) or corticosterone (10 µM). Fluorescent substrates were excited with a pulsed high‐power bluewhite LED (CREE Inc., Racine WI USA) set to 480 nm (ASP^+^) or 450 nm (APP^+^), and the emission was continuously recorded at 609 nm (ASP^+^) or 535 nm (APP^+^) using a high sensitivity photomultiplier tube photo-detector (HAMAMATSU Photonics, Hersching am Ammersee, Germany) system of a reconfigured Sequoia‐Turner 450 fluorometer adapted to an inverted epifluorescence microscope (Olympus IX50, Olympus, Tokyo, Japan). Fluorescent substrates, ethanol, cocaine, and corticosterone were directly applied to the cells by use of a microsuperfusion system (Octaflow™, ALA Scientific Instruments Inc., Farmingdale, NY, USA; set to 5 psi) at the concentrations indicated.

*Data Analysis:* Uptake of APP^+^ and ASP^+^ in cells was analyzed using pClamp10.3, Microsoft Excel and GraphPad Prism. Total area under the curve (A.U.C.) from t_80-150 s_ were analyzed using Brown-Forsythe ANOVA test, with Dunnett's T3 multiple comparisons test versus control.

***In vivo* high-speed chronoamperometry.**

*In vivo* high-speed chronoamperometry was used to examine transporter efficiency by recording clearance of dopamine in real-time (msec). Methods used in these experiments were adapted from our previous publications^4, 10, 25^.

*Electrode Fabrication, Calibration and Micropipette-Electrode Assemblies:* Carbon fiber electrodes used in experiments were fabricated based on established methods^26, 27^ and have been described in detail elsewhere^9, 10^. In brief, a single carbon fiber (30 μm diameter) was sealed in fused silica tubing (Schott North America, Elmsford, NY, USA), and then coated with Nafion (5% solution; Sigma-Aldrich, St. Louis, MO, USA) to prevent passage of anions in extracellular fluid to the carbon fiber^10, 25^. Prior to use *in vivo*, electrodes were calibrated to increasing concentrations of dopamine in the presence of its major metabolite (3,4-dihydroxyphenylacetic acid [DOPAC] *in vitro*. Only electrodes with selectivity ratios greater than 1000:1 for DA over DOPAC, and a linear response (*r^2^* $\geq$ 0.9) to increasing concentrations of dopamine (0 to 3.0 µM) were used in *in vivo* experiments. Once crafted and calibrated, a Nafion-coated carbon fiber electrode was attached to a four-barrel glass micropipette (FHC, Bowdoin, ME, USA) with 200 μm separating the tips. Barrels of the micropipette were filled with either dopamine (200 µM), ethanol (100 mM), cocaine (400 µM), ethanol + cocaine, or vehicle (phosphate-buffered saline, (PBS)). Work in our lab has established that pressure-ejection of ~20 nL of 200 μM dopamine, 200 μm away from the recording electrode, results in signal amplitudes of ~0.5-1.0 μM^10, 25^. Thus, the concentration of neurotransmitter and drug reaching the recording electrode was ~200-400 fold less than the barrel concentration. Additionally, concentrations of ethanol locally applied to dorsal striatum yielded concentrations at the recording site in the range of 0.5-10 mM^1^, a range that is wholly consistent with extracellular brain concentrations reported after systemic administration of 1 g/kg of ethanol^28^. Cocaine was locally applied to dorsal striatum to yield concentrations at the recording site in the range of 1-10 µM, which is consistent with extracellular brain concentrations reported after systemic administration of behaviorally relevant doses of cocaine^29, 30^. Importantly, these concentrations of cocaine robustly inhibit DA clearance, whereas lower concentrations are known to increase DA clearance by trafficking DAT to the plasma membrane^11^. Moreover, cocaine does not have activity at OCT3 at behaviorally/physiologically relevant concentrations^2,8^.

*High-speed Chronoamperometric Readings:* The electrode assembly was lowered into the dorsal striatum (AP + 1.1; ML +/- 1.4; DV -2.25mm to -2.70) of mice anesthetized by intraperitoneal (i.p.) injection (2 ml/kg body weight) of a mixture of urethane (250 mg/kg) and α-chlorolose (25 mg/kg), followed by tracheal intubation to facilitate breathing. Body temperature was maintained at 36–37 °C by a water circulated heating pad, and blood oxygen levels monitored (MouseOximeter, Starr LifeSciences) and maintained above 90%. FAST-16 system (Quanteon, Nicholasville, KY, USA) was used for high-speed chronoamperometric recordings. Oxidation potentials consisted of 100 ms pulses of +0.55 V alternated with 100 ms intervals during which the resting potential was maintained at 0 V. The active electrode voltage was applied with respect to a silver chloride reference electrode placed in the contralateral superficial cortex. Oxidation and reduction currents were digitally integrated during the last 80 ms of each 100 ms voltage pulse. Exogenous DA and drugs were pressure-ejected into striatum using a Picospritzer II (General Valve Corporation). Once reproducible dopamine signals (0.59 ± 0.01 µM, ~4.5 pmol in ~23 nL) were obtained, ethanol (~2 nmol in 100 nl), cocaine (~40 pmol in 136 nl), the combination of ethanol+cocaine (in 136 nl) or PBS (136 nl) was pressure ejected locally into dorsal striatum. Following ethanol, cocaine, ethanol+cocaine, or PBS pressure ejection, dopamine was pressure ejected every 5 min until neurotransmitter clearance time returned to pre-drug values. The T_80_ time course (time it takes for the signal to decline by 80% of the peak signal amplitude), T_20-60_ time course (time to clear between 20% and 60% of the peak signal, i.e. the pseudo linear portion of the descending limb of the DA signal, and peak signal amplitude were analyzed. At the completion of the experiment, an electrolytic lesion was made to mark the placement of the electrode tip. Brains were removed, frozen, and stored at −80 °C for histological analysis. Brains were thawed to −18 °C and sliced into 20 µm thick sections, and stained with thionin for verification of electrode placement.

*Data Analysis:* Separate multifactor (i.e., treatment, genotype, sex) ANOVAs using GraphPad Prism were followed by one-way ANOVAs and Tukey-corrected multiple comparisons to analyze differences in T_20-60_ and T_80_ time courses, and peak signal amplitude between OCT3+/+ and OCT3-/- mice administered either vehicle, EtOH, cocaine, or the combination of EtOH and cocaine.

**Behavior Assays.**

*Drugs.* High performance liquid chromatography grade EtOH (200 proof) and cocaine (EMD Millipore Corporation, Burlington, MD) were diluted in physiologic (0.9%) saline. For CPP and sensitization to the locomotor stimulant effects of these drugs, mice were injected i.p. with physiological saline, saline + EtOH (100 mg/kg), saline + cocaine (3.2 mg/kg), or EtOH (100 mg/kg) + cocaine (3.2 mg/kg) immediately prior to assay. These doses were selected based on our pilot studies demonstrating that these were the highest doses that did not produce statistically significant CPP by themselves in male mice (data not shown). For LORR behavioral assays, mice were injected i.p. with a sedative dose of EtOH, at 3.2 g/kg (base weight per kilogram body weight).

*CPP*. Conditioned place preference (CPP) in rodents, which is often used to model abuse-related drug effects, is shown by a preference for an environment previously paired with a drug compared with an environment previously paired with vehicle^31, 32^. Mice were exposed to an unbiased, one-compartment place-conditioning procedure, with sessions conducted once per day (see^12, 13^). Due to the unbiased design, no exclusion criteria were set, and no mice were excluded. The CPP procedure consisted of three phases**:** habituation (one session), conditioning (eight sessions), and place preference test (one session). The habituation session was intended to reduce the novelty and stress associated with handling, injection, and exposure to the apparatus; thus, for this session, all mice received saline and were placed in the apparatus for 30 minutes on a floor covered with paper. The following 8 days, 30-minute conditioning sessions were held. Conditioning consisted of pairing one floor type with the injection of drug (saline-EtOH, saline-cocaine, or EtOH-cocaine) and the other floor type with the injection of vehicle. After injections, mice were immediately placed in the center of the apparatus. The day after the last conditioning session, the 30-min floor preference test was conducted in mice having received saline. The time spent on the drug-paired floor was subtracted from the time spent on the saline-paired floor, and this difference was used to measure place preference.

*Sensitization*: Sensitization of locomotor activity was measured in the same animals using a procedure similar to that described previously^12, 13^. Sensitization to the locomotor stimulant effects of cocaine and/or EtOH, evidenced by progressively enhanced locomotor responses following repeated administration of cocaine and/or EtOH^33^, was measured during the drug conditioning sessions of the CPP procedure. All sessions were conducted once per day between the hours of 8 AM and 11 AM.

*EtOH-Induced Loss of Righting Reflex.* Loss of righting reflex (LORR) is commonly used to assess the sedative-hypnotic effects of drugs. Here we used male constitutive SERT-/- mice, known to have higher expression of OCT3 than wild-type controls^14^, and constitutive OCT3-/- mice to gain insight into a putative role for OCT3 in the sedative hypnotic effects of EtOH. All mice were injected i.p. with 3.2 g/kg EtOH. Immediately after injection, mice were placed in a supine position in Plexiglass V-shaped containers. LORR was defined as failure to right onto all four paws within 30 seconds of the mouse being placed on its back. Exclusion criterion were mice that did not lose righting within 5 minutes of the injection. No mice were excluded. Recovery of righting reflex (RORR) was achieved when mice could right themselves onto all four paws twice consecutively within 30 seconds of the initial instance of self-righting. Latency of LORR (time elapsed from injection to LORR onset) and duration of LORR (time elapsed from LORR to RORR) were measured.

*Data Analysis:* A three-way ANOVA using GraphPad Prism was used to assess CPP in male and female OCT3+/+ and OCT3-/- animals receiving EtOH, cocaine, or EtOH+cocaine. This analysis was followed with separate two-way ANOVAs to further probe sex*genotype and treatment*genotype interactions. Sensitization to the locomotor stimulant effects of cocaine was first assessed using a four-way repeated measures ANOVA with Greenhouse-Geisser correction, with sex, genotype, treatment, and training day as independent variables. There were no interaction or main effects of sex on sensitization, so male and female data were pooled for further analysis, in which effects of genotype and days were assessed separately for each drug treatment using two-way repeated measures ANOVA’s. All multiple comparisons were conducted using Tukey’s test with p<0.05 considered statistically significant. For the LORR assay, survival analysis, using the Mantel-Cox log-rank test and the Mentel-Haenszel hazard ratio, was used to assess the effect of genotype on duration of EtOH-induced LORR, and latency of LORR onset. The Holmes-Šidák correction for multiple comparisons was used. Statistical significance was defined as *p*<0.05. Statistical analyses were performed with GraphPad Prism.

**Results**

**Concentration Effect Curves for the Ability of Cocaine to Inhibit DA Clearance are Similar Between OCT3+/+ and OCT3-/- Mice.**

SI Figure 1 provides summary data for both the genotype and concentration dependency of the effect of cocaine to inhibit DA T_80_ clearance time 2 min after its application. There were no significant differences between male and female mice, so data were pooled for both sexes. Results demonstrated that there were no significant genotype differences in the maximally effective (OCT3+/+ Emax: 150 ± 13%; OCT3-/- Emax: 154 ± 15%) or half maximally effective (OCT3+/+ EC50: 35 ± 9 pmol; OCT3-/- EC50: 23 ± 16 pmol) amounts of cocaine to inhibit DA clearance. Two-way ANOVA revealed no main effect of genotype (*F* (1, 72) = 0.80, *p* = 0.40), but the expected main effect of cocaine concentration (*F* (3, 72) = 19.19, *p* < 0.001), with no significant interaction (*F* (3, 72) = 0.49, *p* = 0.93).

**Ethanol Potentiates the Ability of Cocaine to Inhibit DA T_20-60_ Clearance Time and Increase DA Signal Amplitude in Dorsal Striatum in an OCT3-Dependent Manner.**

There were no statistically significant main or interaction effects of sex for either T_20-60_ or signal amplitude, so data from males and females were pooled for analyses. There were significant differences in DA clearance time (T_20-60_) among groups administered ethanol, cocaine, or ethanol and cocaine for OCT3+/+ (*p* < 0.0001) and OCT3-/- (*p* < 0.0001) (SI Table 1) mice. T_20-60_ clearance times post-ethanol trended to be longer in OCT3+/+ mice than in OCT3-/- mice (T_20-60_ *p* = 0.09), consistent with OCT3 being a key player in the action of ethanol to inhibit DA uptake. None of the other treatments showed significant differences between genotypes. As expected, a maximally effective pmol amount of cocaine locally applied to dorsal striatum, inhibited DA clearance in both OCT3+/+ (*p* = 0.004) and OCT3-/- (*p* = 0.004) mice (SI Table 1). Consistent with ethanol and cocaine acting at different sites, the ability of ethanol to enhance the DA clearance inhibiting effect of cocaine approached significance in wild-type mice (*p* = 0.06), but not in OCT3-/- animals (*p* = 0.74) (SI Table 1).

In general amplitude was not significantly impacted by genotype and by any of the treatments with one exception.  Consistent with ethanol potentiating the ability of cocaine to inhibit DA clearance, signal amplitude following the combination of ethanol and cocaine in OCT3+/+ mice was significantly greater than those receiving only ethanol (*p* = 0.04) (SI Table 2).  This was not the case in OCT3-/- mice.

There were significant differences between genotypes for baseline (i.e. pre-drug) T_20-60_ values, but not for baseline amplitude values. DA clearance time was significantly longer in OCT3-/- mice (T_20-60_ 18 ± 1 s) than their OCT3 counterparts (T_20-60_ 14 ± 0.9 s) (T_20-60_ *t*_125_ = 2.60 *p* = 0.01). Baseline signal amplitude was not significantly different between genotypes (OCT3+/+ 0.60 ± 0.01 µM; OCT3-/- 0.58 ± 0.01 µM; *t*_125_ = 1.87, *p* = 0.07), which was expected given we controlled the amount of DA pressure-ejected to yield similar signal amplitudes between genotypes. Neither the volume nor pmol amount of DA delivered differed as a function of genotype.

**Discussion**

OCT3 Constitutive KOs: In terms of potential compensation in constitutive OCT3 KO mice, there is currently limited information. We found no difference in DAT protein in dorsal and ventral striatum as well as nucleus accumbens core and shell^2^. Measures of mRNA levels of several related transporters (OCT1, OCT2, OCT3, DAT, NET, SERT) in PMAT KO mice provide no evidence for compensation either^34^. Given substantial redundancy in function and expression of OCT3 and PMAT, it is likely similar findings would be made in OCT3 KO mice. Tissue levels of DA in OCT3 KO mice are reportedly modestly decreased in several brain regions including striatum^35^ consistent with a reduced ability to recapture released DA. To our knowledge, there are no reports of the consequences of constitutive OCT3 KO on other elements of DA signaling. Given the relatively modest neurochemical adaptations, or absence of them, noted so far in constitutive OCT3 KO mice, we are unaware of any that might contribute to current findings. Nonetheless, it will be important for future research to use brain region- and cell-type specific conditional OCT3 depletion strategies, to delve deeper into the mechanistic basis of our present findings.

Ethanol Metabolism: Although we did not measure blood alcohol concentration in OCT3+/+ and OCT3-/- mice, differences in ethanol metabolism between the two genotypes are unlikely. This is based on our findings that, at the time of recovery from LOR, blood alcohol levels were lower in SERT-/- than SERT+/+ mice, thus longer LORR in SERT-/- mice was not an artefact of slower ethanol metabolism^1^, and given present findings, more likely reflective of ethanol’s actions at OCT3. It will be important in future studies to confirm that shorter sleep times in OCT3-/- mice is related to the absence of OCT3 and not to faster ethanol metabolism. However, taken together with data presented here, it appears that LORR is related to level of OCT3 expression.

Future Directions: Collectively, our data provide compelling evidence for OCT3 as a novel and previously unsuspected player in the actions of ethanol to inhibit monoamine uptake. Here we identified OCT3 as an important mediator of the acute effects of ethanol to inhibit DA uptake and produce rewarding effects. These novel findings raise several avenues for exciting future investigations. These include:

1) Investigations of the effect of chronic cocaine and ethanol use in combination or alone. While numerous studies have examined neural consequences of chronic use of each drug independently^36-38^ , few have studied their combined effects^39^. For instance, a recent study of chronic cocaine and ethanol taking in male Rhesus monkeys showed that cocaine decreased D2 receptor availability in control monkeys (not taking ethanol), but D2 receptor availability (measured by positron emission tomography) was not changed in monkeys self-administering cocaine and ethanol^40^. In contrast, D3 receptor function (measured by quinpirole-induced yawning) was increased in monkeys taking cocaine and ethanol, but not in monkeys self-administering only cocaine^40^, underscoring dynamic interplay between cocaine and alcohol in resulting long-term neuroplastic changes. Given present findings pointing to OCT3 as a key-mediator of the effects of ethanol on neurochemistry and behavior, detailed and complex studies will be needed to identify the long term, functional adaptive effects of ethanol and cocaine alone, and in combination, on receptor and transporter availability and neuronal activity.

2) Particularly important for interpretation of our behavioral data, will be to determine if cocaethylene, a psychoactive substance formed in the liver when cocaine and alcohol coexist in blood^41^, either lacks or displays activity at OCT3, as well as acetaldehyde, the main metabolite of ethanol, which is readily self-administered in rodents and could play a role in the rewarding properties of ethanol^42^.

3) It remains unclear how and to what extent ethanol modulates firing rates of monoaminergic neurons *in vivo* following systemic ethanol administration and how it affects monoamine release and clearance in real time. This question could be addressed with genetically encoded sensors for calcium in conjunction with sensors that allow for the detection of extracellular monoamines. Studies are underway to investigate the impact of ethanol on neuronal calcium dynamics and clearance of their respective neurotransmitter.

4) Since behavioral and neurochemical effects of most drugs of abuse, including ethanol and cocaine, differ depending on whether they are administered contingently vs. non-contingently (e.g.^43-45^), it will be important to assess self-administration of ethanol, cocaine and their combination.

5) It will be of fundamental importance to determine how ethanol interacts with OCT3 to inhibit substrate uptake, these efforts will build on the recently published structural basis for OCT3 inhibition^46^. Moreover, the effect of EtOH on OCT3-mediated transport could significantly affect the absorption and elimination of drugs, which further highlights the potential impact of our study.

References

1. Daws LC, Montanez S, Munn JL, Owens WA, Baganz NL, Boyce-Rustay JM *et al.* Ethanol inhibits clearance of brain serotonin by a serotonin transporter-independent mechanism. *J Neurosci* 2006; **26**(24)**:** 6431-6438.

2. Mayer FP, Schmid D, Owens WA, Gould GG, Apuschkin M, Kudlacek O *et al.* An unsuspected role for organic cation transporter 3 in the actions of amphetamine. *Neuropsychopharmacology* 2018; **43**(12)**:** 2408-2417.

3. Baganz N, Horton R, Martin K, Holmes A, Daws LC. Repeated swim impairs serotonin clearance via a corticosterone-sensitive mechanism: Organic cation transporter 3, the smoking gun. *J Neurosci* 2010; **30**(45)**:** 15185-15195.

4. Horton RE, Apple DM, Owens WA, Baganz NL, Cano S, Mitchell NC *et al.* Decynium-22 enhances ssri-induced antidepressant-like effects in mice: Uncovering novel targets to treat depression. *J Neurosci* 2013; **33**(25)**:** 10534-10543.

5. Fraser-Spears R, Krause-Heuer AM, Basiouny M, Mayer FP, Manishimwe R, Wyatt NA *et al.* Comparative analysis of novel decynium-22 analogs to inhibit transport by the low-affinity, high-capacity monoamine transporters, organic cation transporters 2 and 3, and plasma membrane monoamine transporter. *Eur J Pharmacol* 2019; **842:** 351-364.

6. Mitchell NC, Gould GG, Koek W, Daws LC. Ontogeny of sert expression and antidepressant-like response to escitalopram in wild-type and sert mutant mice. *J Pharmacol Exp Ther* 2016; **358**(2)**:** 271-281.

7. Mayer FP, Schmid D, Holy M, Daws LC, Sitte HH. "Polytox" synthetic cathinone abuse: A potential role for organic cation transporter 3 in combined cathinone-induced efflux. *Neurochem Int* 2019; **123:** 7-12.

8. Mayer FP, Luf A, Nagy C, Holy M, Schmid R, Freissmuth M *et al.* Application of a combined approach to identify new psychoactive street drugs and decipher their mechanisms at monoamine transporters. *Curr Top Behav Neurosci* 2017; **32:** 333-350.

9. Williams JM, Owens WA, Turner GH, Saunders C, Dipace C, Blakely RD *et al.* Hypoinsulinemia regulates amphetamine-induced reverse transport of dopamine. *PLoS Biol* 2007; **5**(10)**:** e274.

10. Daws LC, Toney GM. High-speed chronoamperometry to study kinetics and mechanisms for serotonin clearance in vivo. In: Michael AC, Borland LM (eds). *Electrochemical methods for neuroscience*. CRC Press/Taylor & Francis: Boca Raton (FL), 2007.

11. Daws LC, Callaghan PD, Moron JA, Kahlig KM, Shippenberg TS, Javitch JA *et al.* Cocaine increases dopamine uptake and cell surface expression of dopamine transporters. *Biochem Biophys Res Commun* 2002; **290**(5)**:** 1545-1550.

12. Clauss NJ, Koek W, Daws LC. Role of organic cation transporter 3 and plasma membrane monoamine transporter in the rewarding properties and locomotor sensitizing effects of amphetamine in male andfemale mice. *Int J Mol Sci* 2021; **22**(24)**:** 13420.

13. Koek W. Morphine-induced conditioned place preference and effects of morphine pre-exposure in adolescent and adult male c57bl/6j mice. *Psychopharmacology (Berl)* 2016; **233**(11)**:** 2015-2024.

14. Baganz NL, Horton RE, Calderon AS, Owens WA, Munn JL, Watts LT *et al.* Organic cation transporter 3: Keeping the brake on extracellular serotonin in serotonin-transporter-deficient mice. *Proc Natl Acad Sci U S A* 2008; **105**(48)**:** 18976-18981.

15. Fox MA, Andrews AM, Wendland JR, Lesch KP, Holmes A, Murphy DL. A pharmacological analysis of mice with a targeted disruption of the serotonin transporter. *Psychopharmacology (Berl)* 2007; **195**(2)**:** 147-166.

16. Reith ME, Coffey LL. [3h]win 35,428 binding to the dopamine uptake carrier. Ii. Effect of membrane fractionation procedure and freezing. *J Neurosci Methods* 1994; **51**(1)**:** 31-38.

17. Price DA, Owens WA, Gould GG, Frazer A, Roberts JL, Daws LC *et al.* Cb1-independent inhibition of dopamine transporter activity by cannabinoids in mouse dorsal striatum. *J Neurochem* 2007; **101**(2)**:** 389-396.

18. Tejani-Butt SM. [3h]nisoxetine: A radioligand for quantitation of norepinephrine uptake sites by autoradiography or by homogenate binding. *J Pharmacol Exp Ther* 1992; **260**(1)**:** 427-436.

19. D'Amato RJ, Largent BL, Snowman AM, Snyder SH. Selective labeling of serotonin uptake sites in rat brain by [3h]citalopram contrasted to labeling of multiple sites by [3h]imipramine. *J Pharmacol Exp Ther* 1987; **242**(1)**:** 364-371.

20. Mitchell NC, Gould GG, Smolik CM, Koek W, Daws LC. Antidepressant-like drug effects in juvenile and adolescent mice in the tail suspension test: Relationship with hippocampal serotonin and norepinephrine transporter expression and function. *Front Pharmacol* 2013; **4:** 131.

21. Janowsky A, Neve K, Eshleman AJ. Uptake and release of neurotransmitters. *Curr Protoc Neurosci* 2001; **Chapter 7**(1)**:** Unit7 9.

22. Hayer-Zillgen M, Bruss M, Bonisch H. Expression and pharmacological profile of the human organic cation transporters hoct1, hoct2 and hoct3. *Br J Pharmacol* 2002; **136**(6)**:** 829-836.

23. Grundemann D, Schomig E. Gene structures of the human non-neuronal monoamine transporters emt and oct2. *Hum Genet* 2000; **106**(6)**:** 627-635.

24. Duan H, Wang J. Selective transport of monoamine neurotransmitters by human plasma membrane monoamine transporter and organic cation transporter 3. *J Pharmacol Exp Ther* 2010; **335**(3)**:** 743-753.

25. Daws LC, Owens WA, Toney GM. Using high-speed chronoamperometry to measure biogenic amine release and uptake in vivo. In: Bönisch H, Sitte HH (eds). *Neurotransmitter transporters*, vol. 118. Springer New York: New York, NY, 2016, pp 53-81.

26. Gerhardt GA. Rapid chronocoulometric measurements of norepinephrine overflow and clearance in cns tissues. *Voltammetric methods in brain systems*, vol. 27. Humana Press: New Jersey, 1995, pp 117-152.

27. Perez XA, Andrews AM. Chronoamperometry to determine differential reductions in uptake in brain synaptosomes from serotonin transporter knockout mice. *Anal Chem* 2005; **77**(3)**:** 818-826.

28. Robinson DL, Lara JA, Brunner LJ, Gonzales RA. Quantification of ethanol concentrations in the extracellular fluid of the rat brain: In vivo calibration of microdialysis probes. *J Neurochem* 2000; **75**(4)**:** 1685-1693.

29. Fuh MR, Tai YL, Pan WH. Determination of free-form of cocaine in rat brain by liquid chromatography-electrospray mass spectrometry with in vivo microdialysis. *J Chromatogr B Biomed Sci Appl* 2001; **752**(1)**:** 107-114.

30. Nicolaysen LC, Pan HT, Justice JB, Jr. Extracellular cocaine and dopamine concentrations are linearly related in rat striatum. *Brain Res* 1988; **456**(2)**:** 317-323.

31. Bardo MT, Bevins RA. Conditioned place preference: What does it add to our preclinical understanding of drug reward? *Psychopharmacology (Berl)* 2000; **153**(1)**:** 31-43.

32. Cunningham CL, Niehus DR, Malott DH, Prather LK. Genetic differences in the rewarding and activating effects of morphine and ethanol. *Psychopharmacology (Berl)* 1992; **107**(2-3)**:** 385-393.

33. Hemby SE, Jones GH, Justice JB, Jr., Neill DB. Conditioned locomotor activity but not conditioned place preference following intra-accumbens infusions of cocaine. *Psychopharmacology (Berl)* 1992; **106**(3)**:** 330-336.

34. Duan H, Wang J. Impaired monoamine and organic cation uptake in choroid plexus in mice with targeted disruption of the plasma membrane monoamine transporter (slc29a4) gene. *J Biol Chem* 2013; **288**(5)**:** 3535-3544.

35. Vialou V, Balasse L, Callebert J, Launay JM, Giros B, Gautron S. Altered aminergic neurotransmission in the brain of organic cation transporter 3-deficient mice. *J Neurochem* 2008; **106**(3)**:** 1471-1482.

36. Jedema HP, Song X, Aizenstein HJ, Bonner AR, Stein EA, Yang Y *et al.* Long-term cocaine self-administration produces structural brain changes that correlate with altered cognition. *Biol Psychiatry* 2021; **89**(4)**:** 376-385.

37. Pleil KE, Helms CM, Sobus JR, Daunais JB, Grant KA, Kash TL. Effects of chronic alcohol consumption on neuronal function in the non-human primate bnst. *Addict Biol* 2016; **21**(6)**:** 1151-1167.

38. Pleil KE, Lowery-Gionta EG, Crowley NA, Li C, Marcinkiewcz CA, Rose JH *et al.* Effects of chronic ethanol exposure on neuronal function in the prefrontal cortex and extended amygdala. *Neuropharmacology* 2015; **99:** 735-749.

39. McGinn MA, Pantazis CB, Tunstall BJ, Marchette RCN, Carlson ER, Said N *et al.* Drug addiction co-morbidity with alcohol: Neurobiological insights. *Int Rev Neurobiol* 2021; **157:** 409-472.

40. Say FM, Tryhus AM, Epperly PM, Nader SH, Solingapuram Sai KK, George BE *et al.* Effects of chronic cocaine and ethanol self-administration on brain dopamine receptors in a rhesus monkey model of polysubstance abuse. *Addict Biol* 2022; **27**(5)**:** e13219.

41. Perez-Reyes M, Jeffcoat AR. Ethanol/cocaine interaction: Cocaine and cocaethylene plasma concentrations and their relationship to subjective and cardiovascular effects. *Life Sci* 1992; **51**(8)**:** 553-563.

42. Melis M, Diana M, Enrico P, Marinelli M, Brodie MS. Ethanol and acetaldehyde action on central dopamine systems: Mechanisms, modulation, and relationship to stress. *Alcohol* 2009; **43**(7)**:** 531-539.

43. Dworkin SI, Mirkis S, Smith JE. Response-dependent versus response-independent presentation of cocaine: Differences in the lethal effects of the drug. *Psychopharmacology (Berl)* 1995; **117**(3)**:** 262-266.

44. Moolten M, Kornetsky C. Oral self-administration of ethanol and not experimenter-administered ethanol facilitates rewarding electrical brain stimulation. *Alcohol* 1990; **7**(3)**:** 221-225.

45. Porrino LJ, Esposito RU, Seeger TF, Crane AM, Pert A, Sokoloff L. Metabolic mapping of the brain during rewarding self-stimulation. *Science* 1984; **224**(4646)**:** 306-309.

46. Khanppnavar B, Maier J, Herborg F, Gradisch R, Lazzarin E, Luethi D *et al.* Structural basis of organic cation transporter-3 inhibition. *Nat Commun* 2022; **13**(1)**:** 6714.

**Figure S1.** **Concentration effect curves for the ability of cocaine to inhibit DA clearance are similar between OCT3+/+ and OCT3-/- mice.** Data are percent increase in T_80_ time course, 2 minutes post-intrastriatal application of cocaine. There were no significance differences between male and female mice, so data for both sexes were pooled. Maximal inhibition (Emax) and half maximally effective pmol amount of cocaine (EC_50_) values were determined by fitting data to a 4-parameter logistic equation. Emax and EC_50_ values for OCT3+/+ mice were 150 ± 13% and 35 ± 9 pmol, and for OCT3-/- mice, 154 ± 15% and 23 ± 16 pmol. Data are mean and S.E.M. N = 8-12/pmol amount.

**
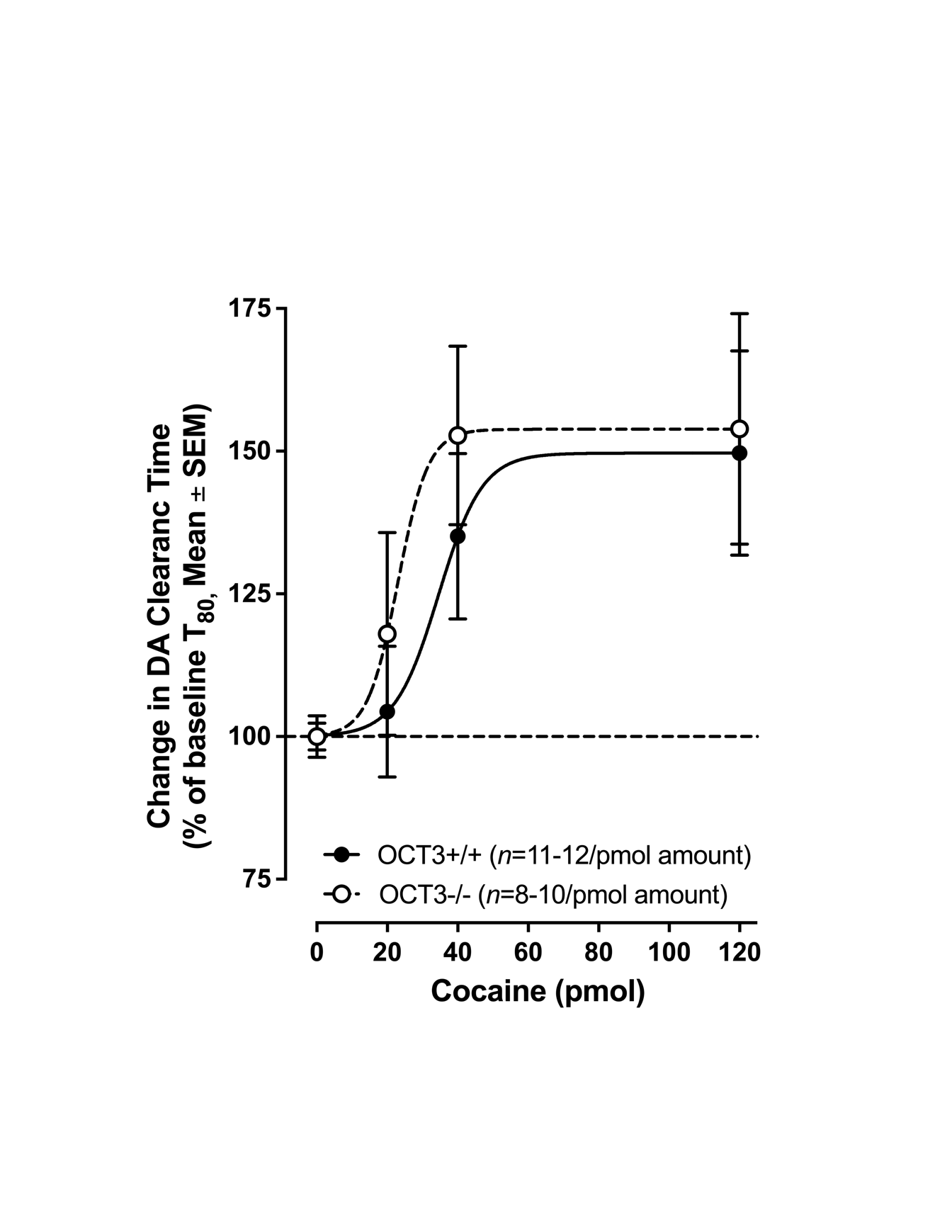
**

**SI Figure 1.**

**Table S1** **Summary data for the effect of cocaine and ethanol to inhibit DA clearance (T_20-60_**) **in dorsal striatum of OCT3+/+ and OCT3-/- mice.** Data are shown as mean and SEM percent change in T_20-60_ value from baseline 2 minutes following administration of drug or vehicle. Data were analyzed using separate 1-way ANOVAs, with Tukey’s correction for post-hoc multiple comparisons. Sample size is shown in parentheses. ** *p* < 0.01, *** *p* < 0.001 vs vehicle; ^##^ *p* < 0.01, ^###^ *p* < 0.001, ^†^ *p* = 0.06 vs ethanol and cocaine; ^$$^ *p* < 0.001 vs cocaine; .

|  | Vehicle | Ethanol | Cocaine | Ethanol & Cocaine |
| --- | --- | --- | --- | --- |
| OCT3+/+ | 103 ± 4 (18) | 123 ± 8 (14)^##^ | 140 ± 8 (16)**/^†^ | 166 ± 11 (16)*** |
| OCT3-/- | 98 ± 4 (16) | 100 ± 6 (17)^###/$$^ | 148 ± 14 (15)** | 159 ± 14 (15)*** |

**SI Table 1.**

**Table S2.** **Summary data for the effect of cocaine and ethanol on DA signal amplitude in dorsal striatum OCT3+/+ and OCT3-/- mice.** Data are shown as mean and SEM percent change in peak amplitude from baseline 2 minutes following administration of drug or vehicle. Data were analyzed using separate 1-way ANOVAs, with Tukey’s correction for post-hoc multiple comparisons. Sample size is shown in parentheses. * *p* < 0.05 vs ethanol.

|  | Vehicle | Ethanol | Cocaine | Ethanol & Cocaine |
| --- | --- | --- | --- | --- |
| OCT3+/+ | 89 ± 7 (18) | 80 ± 8 (14) | 109 ± 14 (16) | 128 ± 16 (16)* |
| OCT3-/- | 77 ± 8 (16) | 70 ± 8 (17) | 106 ± 16 (15) | 110 ± 10 (15) |

**SI Table 2.**

**SI Table 2.**
